# The beneficial effects of exercise in rodents are preserved after detraining: a phenomenon unrelated to GLUT4 expression

**DOI:** 10.1186/1475-2840-9-67

**Published:** 2010-10-28

**Authors:** Alexandre M Lehnen, Natalia M Leguisamo, Graziela H Pinto, Melissa M Markoski, Kátia De Angelis, Ubiratan F Machado, Beatriz Schaan

**Affiliations:** 1 Instituto de Cardiologia do Rio Grande do Sul/Fundação Universitária de Cardiologia, Rio Grande do Sul, Brasil; 2 Universidade Federal de Ciências da Saúde de Porto Alegre, Rio Grande do Sul, Brasil; 3 Laboratório do Movimento Humano, Universidade São Judas Tadeu, São Paulo, Brasil; 4 Instituto de Ciências Biomédicas, Universidade de São Paulo, São Paulo, Brasil; 5 Serviço de Endocrinologia, Hospital de Clínicas de Porto Alegre, Universidade Federal do Rio Grande do Sul, Brasil

## Abstract

**Background:**

Although exercise training has well-known cardiorespiratory and metabolic benefits, low compliance with exercise training programs is a fact, and the harmful effects of physical detraining regarding these adaptations usually go unnoticed. We investigated the effects of exercise detraining on blood pressure, insulin sensitivity, and GLUT4 expression in spontaneously hypertensive rats (SHR) and normotensive Wistar Kyoto rats (WKY).

**Methods:**

Studied animals were randomized into sedentary, trained (treadmill running/5 days a week, 60 min/day for 10 weeks), 1 week of detraining, and 2 weeks of detraining. Blood pressure (tail-cuff system), insulin sensitivity (kITT), and GLUT4 (Western blot) in heart, gastrocnemius and white fat tissue were measured.

**Results:**

Exercise training reduced blood pressure (19%), improved insulin sensitivity (24%), and increased GLUT4 in the heart (+34%); gastrocnemius (+36%) and fat (+22%) in SHR. In WKY no change in either blood pressure or insulin sensitivity were observed, but there was an increase in GLUT4 in the heart (+25%), gastrocnemius (+45%) and fat (+36%) induced by training. Both periods of detraining did not induce any change in neither blood pressure nor insulin sensitivity in SHR and WKY. One-week detraining reduced GLUT4 in SHR (heart: -28%; fat: -23%) and WKY (heart: -19%; fat: -22%); GLUT4 in the gastrocnemius was reduced after a 2-week detraining (SHR: -35%; WKY: -25%). There was a positive correlation between GLUT4 (gastrocnemius) and the maximal velocity in the exercise test (r = 0.60, p = 0.004).

**Conclusions:**

The study findings show that in detraining, despite reversion of the enhanced GLUT4 expression, cardiorespiratory and metabolic beneficial effects of exercise are preserved.

## Background

Systemic arterial hypertension is a clinical condition associated to high morbidity and mortality [1] requiring pharmacological and non-pharmacological treatment. Maintenance of blood pressure (BP) levels within normal ranges greatly reduces its morbidity and mortality [1]. However, compliance with the recommended treatment is low: in a cohort of hypertensive patients, 45% of them discontinued regular follow-up, i.e., only 55% maintained their blood pressure levels within desired ranges [2].

Insulin resistance is commonly associated with arterial hypertension and is *per se *a predictor of cardiovascular risk [3]. It is well known that exercise training improves insulin-stimulated glucose transport, particularly in skeletal muscle, the major site for insulin-stimulated glucose uptake [4]. This effect is mediated by increased translocation of GLUT4 from cytoplasm to plasma membrane [5]. Increased levels of GLUT4 in the plasma membrane due to an increase in gene expression and/or translocation are directly correlated with improved insulin sensitivity [6]. Knowing the underlying mechanisms for this benefit, as well as their reversibility, may help devising new preventive and therapeutic actions for systemic arterial hypertension and insulin resistance.

However, low compliance with exercise training programs is a fact [7]. The harmful effects of physical detraining regarding cardiorespiratory and metabolic adaptations usually go unnoticed. Cardiac output, resting heart rate and peripheral vascular resistance return to pre-training levels within 5 weeks of physical detraining [8]. As for metabolic changes, it has been evidenced that 48-h [9] to 1-week [10] cessation of exercise training reduces the levels of GLUT4 gene expression in normotensive rats. Therefore, it is necessary to identify the physiological consequences of such interference, as well as the possible mechanisms involved, in hypertensive subjects. Thus, 1 and 2 weeks of detraining periods are appropriate for investigating the association between cardiorespiratory variables and GLUT4 expression downregulation. There are no studies in literature investigating the effect of physical detraining on GLUT4 gene expression in the plasma membrane of hypertensive individuals. In this aspect, the spontaneously hypertensive rats (SHR) is an useful model, because it presents hypertension (with spontaneous systemic development as observed in humans) and glucose metabolism disturbs [11]. The objective of the present study was to assess the effects of physical detraining (1 and 2 weeks) on metabolic (insulin sensitivity and GLUT4 gene expression) and cardiorespiratory variables (BP and functional capacity) in spontaneously hypertensive rats.

## Methods

All procedures of animal experimentation followed the Guide for the Care and Use of Laboratory Animals [12]. The study was approved by the Research Ethics Committee of Instituto de Cardiologia do RS (# UP:3859.06).

### Animals

A total of 64 male rats aged 6 months were studied, of which 32 were spontaneously hypertensive (SHR) and 32 were Wistar Kyoto (WKY) rats. SHR and WKY rats were randomized (n = 8 per group) according to the proposed protocol of exercise training: SN (sedentary normotensive), SH (sedentary hypertensive), TN (trained normotensive), TH (trained hypertensive), DN1 (1-week-detrained normotensives), DH1 (1-week-detrained hypertensives), DN2 (2-week-detrained normotensives) and DH2 (2-week-detrained hypertensives).

Animals were allowed free access to water and standard rodent chow diet (Nuvilab CR-1, Nuvital, Curitiba, Brazil)(12-hour light/12-hour dark cycle, 6 a.m./6 p.m.) and temperature (23 ± 2°C) conditions.

### Blood pressure

BP was measured using a tail-cuff system (Model 229, IITC Life Science Inc.) before, during (5^th ^week), and after the exercise training (SN, SH, TN and TH groups) or the detraining (DN1, DH1, DN2 and DH2 groups) periods. All animals were placed in a restrainer for 15 minutes, a cuff was attached to their tail and BP was then recorded. They previously underwent an adaptation period to get used to this procedure that was repeated in three days. BP measures obtained from day 4 were considered valid.

### Insulin tolerance test

An insulin tolerance test was performed as proposed in the literature [13,14] using commercially available regular insulin (Humulin, Eli Lilly, São Paulo, Brazil). The insulin tolerance test of trained groups (TN and TH) was performed 24 hours after the last exercise bout, in order to avoid exercise-induced acute effects [15] and at the same time of the day in their respective control groups (SN and SH). Detrained groups (DN1, DN2, DH1 and DH2) were evaluated at the end of their respective periods. After 3 h of food deprivation, the rats were anesthetized (50 mL.kg^-1 ^ketamine and 20 mL.kg^-1 ^xylazine; 0.2 mL/100 g), and then insulin (0.75 U/kg) was administered into the penian vein. This test comprises 6 blood glucose measures using test strips (Accu-check Advantage, Roche, Mannheim, Germany) at baseline (before insulin administration), 4, 8, 12, 16, and 20 minutes post-insulin application. Glucose measures were then converted into natural logarithm (Ln); the slope was calculated using linear regression [time × Ln (glucose)] and multiplied by 100 to obtain the glucose decay constant rate during insulin tolerance test (kITT) per minute (%.min^-1^).

### Maximal exercise test

Functional capacity was measured by the maximal exercise test (ET) as described in the literature [16]. First, all rats were submitted to an adaptation period on the treadmill (Inbramed, Brazil) at a speed of 0.3 km/h during 15 minutes for three consecutive days. The maximal exercise test was then performed individually at an initial speed of 0.3 km/h with increments of 0.3 km/h every 3 minutes.

### Exercise training and detraining

The dynamic aerobic exercise training, prescribed based on ET, was performed at low-moderate intensity (~ 50% to 70% maximal running speed of the ET) for 1 hour a day (7 pm to 8 pm), 5 days a week for 10 weeks, with a gradual increase in speed from 0.6 to 1.2 km/hr. After a 10-week training, detrained groups were confined in plastic cages for one-week or two-week detraining periods.

### Tissue preparation and euthanasia

Tissue samples were removed from trained animals 40 hours after the last exercise bout, in order to avoid interferences in GLUT4 expression [17]; and from detrained animals at the end of their respective periods (at 9-12 am). For this procedure, rats were normally fed and then anesthetized with a high dose of pentobarbital sodium (100 mg/kg). The gastrocnemius muscle was entirely removed from the right hind limb, and a transversal red and white mixed segment was processed to obtain a homogenate. After the removal of the white fat tissue (epididymal), thoracotomy was performed, and immediately the heart was entirely removed, before respiratory arrest. Cardiac muscle homogenate was obtained from the whole heart.

All tissue specimens were homogenized as described by Machado *et al*. [18]. Briefly, fat tissue was homogenized with a Polytron Homogeneizer (Marconi) 20,000 rpm for 30 s in buffer (7.4 pH, 10 mmoL/L Tris-HCl, 1 mmoL/L EDTA and 250 mmol/L sucrose) at a weight: volume ratio of 1:4 and centrifuged at 2,000 g for 15 minutes at 4°C. The supernatant containing free-fat extract (FFE) was separated and centrifuged at 12,000 g for 15 minutes at 4°C. The pellet was resuspended in 1 mL buffer corresponding to the plasma membrane (PM) fraction.

The muscle was homogenized in the same buffer and centrifuged at 1,000 g for 10 minutes. The supernatant was separated, and the pellet was resuspended into 1/3 of the initial volume and centrifuged at 150,000 g for 75 minutes. The pellet was again resuspended into 1 mL buffer forming the final sample corresponding to the membrane fraction.

### Western blot

GLUT4 gene expression was determined by Western blot. Protein concentration was determined using the Bradford method. Specimens, 75 μg of each, were solubilized in NuPAGE buffer (Invitrogen, Carlsbad, USA). For electrophoresis separation (SDS-PAGE) protein extracts were applied into 10% denaturing polyacrilamide/bis-acrylamide gel (Invitrogen, Carlsbad, USA) with a molecular weight marker (BenchMark, Invitrogen, Burlington, USA). Proteins were then transferred to nitrocellulose membrane (Ge Healthcare, New York, USA). Non-specific protein bindings were reduced through incubation (1 h) with a block buffer (5% non-fat skim milk powder in PBS at pH 7.4). The nitrocelulose membrane was incubated with anti-GLUT4 polyclonal antibody (#07-1404, Millipore, Billerica, USA) titrated to 1:2,000 overnight at 4°C. The membrane was washed (0.05% PBS/Tween 20, 1 × 15 min, 3 × 5 min) and incubated (1 h) with a secondary antibody, 1:12.000 goat anti-rabbit IgG (#12-349, Millipore, Billerica, USA). The membrane was rewashed and incubated in the dark with peroxidase substrate (ECL kit, GE Healthcare, New York, USA) for 3 min and exposed to an ultrasensitive radiographic film (Kodak, Frankfurt, Germany) for 1 h. Blot intensity was quantified by optic densitometry using Scion Image software, and results were expressed as arbitrary units (AU/μg protein).

### Statistical analysis

The results were compared using analysis of variance (one-way or repeated measures ANOVA as applicable; p < 0.05) and Tukey's post-hoc test and described as means and standard deviations. The comparisons were made between groups of the same experimental model. Pearson correlation was used to study the association between variables.

## Results and Discussion

Tables 1 and 2 show baseline characteristics of the studied animals and the effects of training or detraining on these variables in WKY rats (Table 1) and SHR (Table 2). At the study entry the body weight, insulin sensitivity and maximal exercise test results were similar between groups and all rats had either high (SHR) or normal (WKY) blood pressure levels as expected.

**Table 1:** Effect of exercise training and detraining in normotensive rats

	SN		TN		DN1		DN2	
	
	Initial	Final	Initial	Final	Initial	Final	Initial	Final
BW(g)	300 ± 10	354 ± 29†	291 ± 19	363 ± 16†	288 ± 13	357 ± 17†	294 ± 12	366 ± 20†
BP(mmHg)	119 ± 9	121 ± 6	121 ± 11	117 ± 7	123 ± 7	118 ± 5	116 ± 12	113 ± 7
kITT(%/min)	4.1 ± 0.4	3.9 ± 0.4	3.9 ± 0.7	4.2 ± 0.5	4.0 ± 0.6	3.8 ± 0.5	4.1 ± 0.4	4.0 ± 0.3
ET (km/h)	1.2 ± 0.2	1.3 ± 0.2	1.3 ± 0.2	2.4 ± 0.3*†	1.3 ± 0.2	2.0 ± 0.3*†	1.2 ± 0.1	2.2 ± 0.3*†

SN = sedentary normotensive, TN = trained normotensive, DN1 = 1-week-detrained normotensives, DN2 = 2-week-detrained normotensives. BW = body weight, BP = blood pressure, kITT = glucose decay constant rate during insulin tolerance test, ET = maximal velocity in the exercise test. n = 8 for all groups. * p < 0.05 vs. S, † p < 0.05 vs. baseline. Repeated measures ANOVA (Tukey's post-hoc test).

**Table 2:** Effect of exercise training and detraining in hypertensive rats

	SH		TH		DH1		DH2	
	
	Initial	Final	Initial	Final	Initial	Final	Initial	Final
BW(g)	316 ± 24	366 ± 21†	313 ± 22	358 ± 12†	335 ± 16	376 ± 13†	324 ± 19	374 ± 10†
BP(mmHg)	182 ± 8	186 ± 7	188 ± 12	153 ± 9*†	183 ± 10	156 ± 11*†	178 ± 17	151 ± 12*†
kITT(%/min)	3.8 ± 0.7	3.6 ± 0.6	3.8 ± 0.6	4.7 ± 0.5*†	3.7 ± 0.4	4.5 ± 0.4*†	3.9 ± 0.3	4.6 ± 0.4*†
ET (km/h)	1.3 ± 0.2	1.2 ± 0.3	1.4 ± 0.1	2.5 ± 0.2*†	1.3 ± 0.2	2.1 ± 0.3*†	1.4 ± 0.2	2.0 ± 0.2*†

SH = sedentary hypertensive, TH = trained hypertensive, DH1 = 1-week-detrained hypertensives, DH2 = 2-week-detrained hypertensives. BW = body weight, BP = blood pressure, kITT = glucose decay constant rate during insulin tolerance test, ET = maximal velocity in the exercise test. n = 8 for all groups. * p < 0.05 vs. S, † p < 0.05 vs. baseline. Repeated measures ANOVA (Tukey's post-hoc test).

Among normotensive rats, the 10-week aerobic training did not induce any changes in blood pressure (Table 1). However, these animals had a body weight gain of ~25% over time (p < 0.001) regardless of baseline values. There was no change in insulin sensitivity over time post-training (TN) or detraining (DN1 and DN2). Functional capacity, evaluated by the maximal velocity obtained in the ET, increased about 85% in trained normotensive as compared to sedentary normotensive rats (p < 0.001). There was no functional capacity impairment after 1 or 2 weeks of detraining.

Among hypertensive rats, the 10-week training induced a reduction of blood pressure (~19% compared to baseline values) and an improvement of insulin sensitivity (~24% compared to baseline values, Table 2). Body weight increased ~15% over time (p < 0.001). Regarding exercise capacity, trained SHR presented ~79% enhancement in ET performance in relation to sedentary SHR (p < 0.001). All of these improvements were not affected by both detraining periods evaluated (1 or 2 weeks). Both studied periods of detraining were found to be insufficient to revert cardiorespiratory and metabolic changes induced by training as no difference was seen between variables after 10-week training and those measured within 1 week and 2 weeks after exercise cessation.

**Figure 1:**
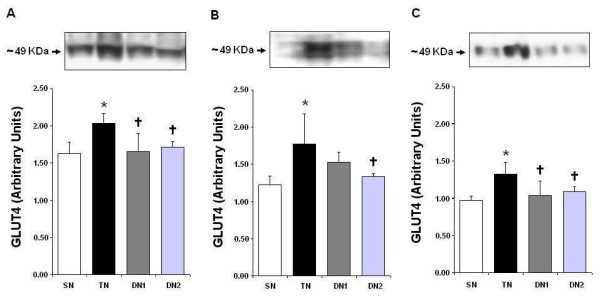
**Analysis of the levels of GLUT4 gene expression in the plasma membrane in normotensive rats. **Western Blot bands and their related quantitative analyses are presented. Panel A, heart; Panel B, gastrocnemius muscle; and Panel C, white fat tissue. n = 5 for all groups. SN = sedentary normotensive, TN = trained normotensive, DN1 = 1-week-detrained normotensives, DN2 = 2-week-detrained normotensives. * p < 0.05 vs. SN; † p < 0.05 vs. TN. One-way ANOVA (Tukey's post-hoc test).

Figure 1 shows the levels of GLUT4 gene expression in the heart (A), gastrocnemius muscle (B), and white fat tissue (C) in normotensive rats. Compared to sedentary rats, exercise training induced 25%, 45%, and 36% increase in GLUT4 gene expression in these tissues, respectively. One-week detraining caused a reduction in GLUT4 gene expression in the heart (-19%) and white fat tissue (-22%) compared to the levels observed after a 10-week aerobic training. However, no reduction (-13%) of gastrocnemius muscle GLUT4 concentration was observed after 1 week of detraining (p = 0.330). When compared to the trained group, a difference was found (p = 0.050) in the gastrocnemius muscle within 2 weeks of detraining with a 25% reduction of GLUT4 gene expression compared to that obtained immediately after the training period (Figure 1).

**Figure 2:**
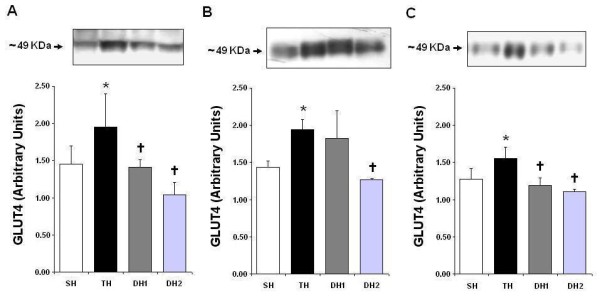
**Analysis of the levels of GLUT4 gene expression in the plasma membrane in hypertensive rats. **Western Blot bands and their related quantitative analyses are presented. Panel A, heart; Panel B, gastrocnemius muscle; and Panel C, white fat tissue. n = 5 for all groups. SH = sedentary hypertensive, TH = trained hypertensive, DH1 = 1-week-detrained hypertensives, DH2 = 2-week-detrained hypertensives. * p < 0.05 vs. SH; † p < 0.05 vs. TH. One-way ANOVA (Tukey's post-hoc test).

Figure 2 shows the levels of GLUT4 gene expression in the heart (A), gastrocnemius muscle (B), and white fat tissue (C) in hypertensive rats. Similar to what was observed in normotensive animals, 10 weeks of training induced greater GLUT4 gene expression in trained SHR compared to sedentary SHR. There was an increase in protein expression of 34% (p = 0.049) in the heart, 36% (p = 0.014) in the gastrocnemius muscle, and 22% (p = 0.008) in white fat tissue. These benefits were reverted within one week of detraining with a reduction of 28% (p = 0.030) in the heart and 23% (p = 0.001) in white fat tissue. As observed in normotensive animals, 1 week of detraining was not sufficient for gastrocnemius GLUT4 levels return to pre-training levels, which remained at a concentration similar to that seen in the trained group. However, there was a 36% reduction (p = 0.001) in muscle GLUT4 expression after 2 weeks of detraining, with loss of the benefits induced by the 10-week aerobic training.

**Figure 3:**
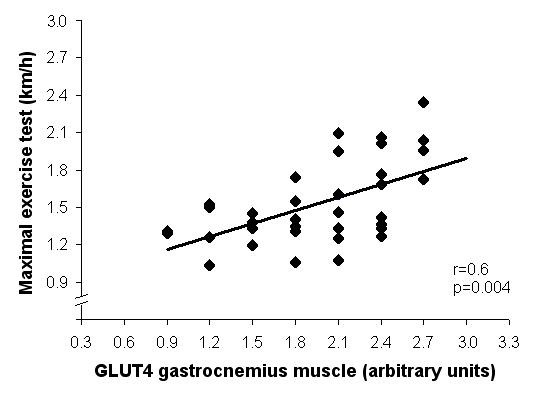
Scattergram plotting GLUT4 expression gastrocnemius muscle (arbritary units) levels and maximal exercise test (km/h) involving all studied groups.

There was a positive correlation between GLUT4 gene expression in the gastrocnemius and the maximal velocity obtained in the exercise test (r = 0.60, p = 0.004; Figure 3).

The present study shows that 1 or 2 weeks of exercise detraining could revert the benefits of raised GLUT4 gene expression determined by aerobic exercise training in hypertensive and normotensive rats, although the positive cardiorespiratory (reduction in blood pressure and increase in functional capacity) and insulin sensitivity effects remained for up to 2 weeks in hypertensive animals.

Our study showed the effects of exercise training in an animal model of hypertension: improved insulin sensitivity, increased GLUT4 gene expression, and reduced blood pressure. Among these beneficial effects, only increased GLUT4 gene expression was also observed in normotensive animals. A 10-week aerobic training reduced systolic blood pressure in hypertensive animals (~19%), as expected [19]. Training-induced hypotensive effect is determined by physiological adaptations in the heart and vascular system, such as increased sympathetic baroreflex sensitivity in the heart, that is usually impaired by the hypertensive status [20], increased capillary density [21], and increased endothelial nitric oxide synthase (eNOS) levels causing endothelium-dependent vasodilatation [22].

Both 1 week and 2 weeks of detraining were not sufficient to revert cardiorespiratory benefits induced by 10 weeks of training in hypertensive rats, which could be in part attributed to a delayed loss of trained-induced resting bradycardia after a detraining period, as previous reported in other studies [23]. Additionally, some authors demonstrated that the resting cardiac output was decreased in trained SHR, and it returned to sedentary SHR values only after 5 weeks of detraining. This detraining effect was most likely caused by an increase in resting heart rate [8]. Mostarda *et al*. [24] described similar results in diabetic animals: no reversal of hemodynamic benefits (BP, heart rate, sympathetic and vagal tonus) within 3 weeks of detraining. In constrast, Bocalini *et al*. [25] demonstrated that 2 weeks of detraining was sufficient to reverse some beneficial cardiovascular effects, including variables related to cardiac hypertrophy. The discrepancy between the results are probably caused by different type and duration of training periods.

The maximal exercise test showed that the functional capacity improved ~85% and ~79% in normotensive and hypertensive animals, respectively, which is consistent with what was described by other authors that directly evaluated the maximum oxygen consumption VO_2 _max [26]. Kemi *et al*. [23] studying Sprague-Dawley female rats reported 50% loss of VO_2 _max within 2 weeks of detraining, which remained only 5% higher than the VO_2 _max in the control group within 4 weeks of detraining. Considering that the VO_2 _max is determined by cardiac (cardiac output) and peripheral (arterio-venous oxygen difference) factors, this response can be a result of reduced ability of myocardial contraction and failed relaxation of heart fibers. Oxygen supply to the myocardium also directly affects functional capacity. In this aspect, Marini *et al*. [27] demonstrated that only after 4 weeks of detraining there was a change in capillary angiogenesis leading to reduced myocardial blood supply. Furthermore, the correlation between maximal exercise test velocity and gatrocnemius GLUT4 expression observed in the present study suggests that changes (improve after training and slight decrease after detraining) in performance could be attributed in part to changes in glucose disposal to oxidation, and consequently in energy production in skeletal muscle.

In the present study, we also demonstrated increased GLUT4 protein expression in skeletal muscle, in addition to increased expression in the heart and white fat tissue, further supporting the finding of increased insulin sensitivity observed in trained hypertensive rats. Although the insulin sensitivity remained unchanged, increased GLUT4 was also seen in trained normotensive rats. Some mechanisms may explain the increased levels of GLUT4 in response to exercise training. Muscle contraction increases AMPK levels and this kinase induces the translocation of GLUT4 storage vesicles (GSVs) to the plasma membrane [28]. In addition, AMPK can phosphorylate HDAC5 protein, which is then separated from the enhancer, thus favoring GLUT4 expression [29]. In parallel to AMPK-mediated induction, there is evidence that indicate a positive relationship between calcium concentration and increased glucose transport into the cell as chronic effects [30].

Our study showed that exercise training increased insulin sensitivity and myocardial GLUT4 expression in parallel with the reduction of blood pressure in hypertensive rats. These results could be related to potential benefits on clinical pathologic conditions, such as diabetic cardiomyopathy, since it was previously shown that changes in myocardial metabolism contributes to myocardial dysfunction in this condition [31].

Cessation of exercise training for 1 week was sufficient to reduce the levels of GLUT4 expression in the heart and white fat tissue in both SHR and normotensive rats. Our findings are corroborated by Neufer *et al*. study [10], that studied normotensive rats, showing that 1 week of detraining was sufficient to revert the beneficial effects on the levels of GLUT4 in soleus and red vastus lateralis induced by aerobic training. Reynolds *et al*. [32] tested two training protocols (swimming and treadmill) in Wistar rats followed by 1 day and 2 days of detraining. Reversal of GLUT4 in epitrochlearis muscle was observed within 48 h regardless of the training modality. The half-life of GLUT4 protein is short and it has been described as ranging between 8-10 h [17], which corroborates these results. However, it is important to emphasize that most of the studies on the effects of detraining usually involve athletes and/or healthy subjects. Only few studies evaluated the effects of detraining in a disease condition. In this aspect the present study was the first to describe this data in SHR.

In both animal models, the levels of muscle GLUT4 protein returned to baseline only after 2 weeks of detraining. This muscle contains oxidative and glycolytic fibers and a mixed segment of gastrocnemius muscle containing both types of fibers was examined in our study. A previous study [10] demonstrated no increase in GLUT4 in vastus lateralis muscle specimens containing glycolytic fibers. It is possible that muscle tissue with predominantly more white than oxidative fibers modulates GLUT4 in a different manner. Another possible explanation is that during exercise training glycolytic fibers were less activated than oxidative ones and therefore had different adaptations.

It is noteworthy that our study showed a reduction in GLUT4 levels in hypertensive rats within 1 week and 2 weeks of detraining while their insulin tolerance test results remained unchanged during the same period. In SHR, GLUT4 in plasma membrane of myocytes increases with age as part of a compensation mechanism due to abnormalities characteristic of hypertensive status [11], and this fact suggests that GLUT4 gene expression does not have a key role in the development of insulin resistance in these animals. For this reason, a direct relationship between insulin tolerance test results and the levels of GLUT4 gene expression in the sample studied cannot be established. Another explanation is that different levels of GLUT4 in these tissues could be an *a priori *adaptation to the uptake of insulin-dependent glucose in the insulin tolerance test.

It is known that obese-induced insulin resistance involves a GLUT4-related reduction in insulin-mediated peripheral [33] and myocardial glucose utilization [31]; the later highly related to myocardial dysfunction [31]. Besides, hypertension is related to insulin resistance, a feature that can be genetically induced [34], with further environmental modulation such as salt intake [34,35], and also involves impaired GLUT4 expression, as observed in some models of hypertension [34,35]. In this broad-spectrum of GLUT4-related impaired glucose utilization, the present data of exercise-induced improvement of GLUT4 expression in hypertensive rats reveal a mechanism by which glycemic homeostasis and myocardial function can ameliorate in parallel. On the other hand, the preservation of cardiorespiratory and metabolic beneficial effects after detraining observed here were not observed in detrained female Wistar rats submitted to a different training protocol [25], once more reinforcing the utmost role of the interaction between genetic and environmental conditions upon these regulations.

## Conclusions

Our results show that changes in the levels of GLUT4 gene expression in SHR are more sensitive to detraining that those associated with blood pressure reduction, since it remained decreased after training cessation. A possible explanation is that hemodynamic effects especially due to arterial hypertension involve different morphophysiological mechanisms and therefore would have a longer impact compared to changes of GLUT4 gene expression. Another assumption would be that GLUT4 levels do not necessarily have a major role in the development of insulin resistance associated with hypertension in SHR as reported in previous studies [11].

The association between muscle contraction and increased GLUT4 levels in the plasma membrane has been well established. However, the mechanisms involved in the modulation of these benefits have yet to be clarified. Once these mechanisms are fully understood and well established, the knowledge on their reversibility and how long it takes for each signaling pathway to loss adaptation induced by exercise training may help devising new preventive and therapeutic actions for systemic arterial hypertension and insulin resistance.

## Competing interests

The authors declare that they have no competing interests.

## Authors' contributions

AML was involved in conception and design of the study, data collection, data analysis and interpretation, as well as drafting and editing the final document for publication. NML and GHP were equally involved in data collection (exercise training, tissue and blood collection, molecular analysis). MMM was involved in data collection (sample preparing, molecular analysis), data analysis and interpretation. KDA was involved in data analysis and interpretation, as well as interpretation and final document writing for publication. UFM was involved in conception and design of the study, data analysis and interpretation, as well as reviewing all parts of the final document for publication. BS was involved in conception and design of the study, data analysis and interpretation, as well as writing, drafting and editing the final document for publication. All authors read and approved the final manuscript.
